# Enhancement of Polymerase Activity of the Large Fragment in DNA Polymerase I from* Geobacillus stearothermophilus* by Site-Directed Mutagenesis at the Active Site

**DOI:** 10.1155/2016/2906484

**Published:** 2016-11-17

**Authors:** Yi Ma, Beilei Zhang, Meng Wang, Yanghui Ou, Jufang Wang, Shan Li

**Affiliations:** ^1^School of Bioscience & Bioengineering, South China University of Technology, Guangzhou 510006, China; ^2^Guangdong Province Key Laboratory of Fermentation and Enzyme Engineering, South China University of Technology, Guangzhou 510006, China

## Abstract

The large fragment of DNA polymerase I from* Geobacillus stearothermophilus* GIM1.543 (Bst DNA polymerase) with 5′-3′ DNA polymerase activity while in absence of 5′-3′ exonuclease activity possesses high thermal stability and polymerase activity. Bst DNA polymerase was employed in isothermal multiple self-matching initiated amplification (IMSA) which amplified the interest sequence with high selectivity and was widely applied in the rapid detection of human epidemic diseases. However, the detailed information of commercial Bst DNA polymerase is unpublished and well protected by patents, which makes the high price of commercial kits. In this study, wild-type Bst DNA polymerase (WT) and substitution mutations for improving the efficiency of DNA polymerization were expressed and purified in* E. coli*. Site-directed substitutions of four conserved residues (Gly^310^, Arg^412^, Lys^416^, and Asp^540^) in the activity site of Bst DNA polymerase influenced efficiency of polymerizing dNTPs. The substitution of residue Gly^310^ by alanine or leucine and residue Asp^540^ by glutamic acid increased the efficiency of polymerase activity. All mutants with higher polymerizing efficiency were employed to complete the rapid detection of EV71-associated hand, foot, and mouth disease (HFMD) by IMSA approach with relatively shorter period which is suitable for the primary diagnostics setting in rural and underdeveloped areas.

## 1. Introduction

Nucleic acid amplification techniques are widely used in diagnosis of infectious diseases, genetic traits, and other clinical medics in application-oriented fields. However, the traditional PCR procedure is time consuming and requires expensive special equipment which is rarely available in rural and underdeveloped areas. Recently Notomi et al. developed a rapid detection of nucleic acid bases, loop-mediated isothermal amplification (LAMP), which with high sensitivity and specificity successfully accomplished the amplification of the target DNA sequence at constant temperature in water bath for one hour neglecting normal three-temperature cycles [1, 2]. LAMP technique has been widely used in the virus detection for infectious diseases, such as avian influenza [3], HFMD [4], Dengue Virus [5], the human immunodeficiency virus [6], and Ebola virus [7]. Presently, a novel and simple colorimetric isothermal multiple self-matching initiated amplification (IMSA) was developed to achieve rapid detection of EV71 in the early phase of HFMD through visual color change by addition of hydroxyl naphthol blue dye [8]. Similar to the LAMP assay, the IMSA assay is an* in vivo* nucleic acid amplification technique depending on Bst DNA polymerase, dNTPs, magnesium ion betaine, and three pairs of primers consisting of one pair of stem primers (SteF and SteR), one pair of outer primers (DsF and DsR), and one pair of inner primers (FIT and RIT). Compared to the LAMP assay, primer design for IMSA assay has its own distinct features which significantly reduce the detection limits of LAMP assay. In order to make quantitative analysis accurately, the IMSA assay generated a fluorescence increase in positive samples allowing real-time monitoring detection.

Since Kornberg discovered and studied DNA polymerase I in detail from* Escherichia coli* in 1958 [9], various DNA polymerases such as DNA polymerases *α* and *β* and RNA polymerase have been isolated and characterized from both prokaryotic and eukaryotic organisms [10, 11]. The indispensable Bst DNA polymerase I in the isothermal amplification was widely applied and played a key role in clinical rapid detection of virus infections. Similar to other DNA polymerase I, Bst DNA polymerase from* Bacillus stearothermophilus* consisted of three independent domains which perform biological function individually: (I) 5′-3′ exonuclease activity, (II) 5′-3′ DNA polymerase activity, and (III) 3′-5′ exonuclease activity [12]. The domains II and III are located in the C-terminus of Bst DNA polymerase, designated as the large fragment (LF) [13]. The full-length of Bst DNA polymerase I gene consisted of 2634-base-pair, while the truncated LF gene devoid of the 5′-terminal 876-base-pair could be translated into a predicated 67.1 kD DNA polymerase with C-terminal poly (His)_6_ which lacks the inherent 5′-3′ proofreading exonuclease activity and was extremely efficient in polymerizing dNTPs in PCR [14]. Since Bst DNA polymerase meets all requirements for high sensitivity, polymerization activity, and thermal stability, it is extensively applied in isothermal amplification procedure of rapid detection. However, Bst DNA polymerase is only available, popular in some international companies as New England Biolabs at high price. Therefore, it is urgent to design, produce, and purify a new and efficient Bst DNA polymerase to satisfy growing demand of less developed area. In this study, we cloned the gene of Bst DNA polymerase I from a new strain (*Geobacillus stearothermophilus* GIM1.543) and make various site-directed substitutions of four conserved residues in the polymerase active site which is important in binding the DNA primer terminus and dNTP to catalyze the polymerase reaction [15, 16]. Three mutants showed higher polymerization activity than the commercial Bst DNA polymerase and, consequently, would lay the roots for promoting its wide application.

## 2. Materials and Methods

### 2.1. Strains, Plasmids, Enzymes, and Reagents

*Geobacillus stearothermophilus* was purchased from Microbial Culture Collection Center of Guangdong Institute of Microbiology, China. Competent* E. coli* cells* DH5α* and BL21 (DE3) were purchased from TIANGEN Biotech (Beijing, China). Plasmid pET21a was preserved by our own laboratory. Restriction endonucleases* Nde* I and* Xho* I were purchased from ThermoFisher Scientific Co., Ltd. (Shanghai, China). Reagents related to Bst DNA pol LF gene cloning and site-directed mutagenesis were purchased from TaKaRa Biotechnology Co., Ltd. (Dalian, China). The HisTrap^FF^ column from GE Healthcare (Piscataway, NJ, USA) was used for proteins purification. The chemicals used for HPLC were obtained from Sigma-Aldrich Chemical (St. Louis, USA). All chemicals used in this study were of analytical grade.

### 2.2. Construction of Expression Vector

Genome DNA from* Geobacillus stearothermophilus* was prepared with a genome extraction kit (Sangon, Shanghai, China) according to manufacturer's handbook. The coding region of LF in Bst DNA polymerase (Bst pol LF) using PrimeSTAR HS DNA polymerase was amplified with the following primers: the forward primer was 5′-CTGTTC**CATATG**GAAGGCGAAAAGCCGCTCGCC-3′ and the reverse primer was 5′-CCG**CTCGAG**TTTGGCGTCGTACCACGTC-3′. Restriction sites* Nde* I and* Xho* I used for subsequent amplification are shown in bold and underline. PCR was implemented under the following condition: initial heating at 95°C for 5 min; 30 cycles of 95°C for 30 s, 56°C for 1 min, and 72°C for 10 min, followed by an extension step at 72°C for 5 min. The PCR product of 1758 bp DNA fragment was separated by 1.5% agarose gel electrophoresis and purified using a DNA gel extraction kit (Sangon, Shanghai). Purified PCR product was digested with* Nde* I and* Xho* I and ligated into pET21a vector (Novagen, Madison) yielding the plasmid pET21a-Bst-LF-H_6_ with one poly (His)_6_ tag at C-terminus and transformed into* E. coli* DH5*α* cells. Recombinant DH5*α* cells were spread on Luria–Bertani (LB) agar plates containing 50 ng/*μ*L of kanamycin and incubated at 37°C overnight. The positive clones were confirmed by colony PCR and DNA sequence analysis. The sequence similarity of Bst DNA pol LF was checked by the BLAST program.

### 2.3. Mutagenesis Experiment

Multiple protein sequences including WT target protein sequence were aligned and analyzed. Several amino acid residues were selected as mutation sites shown in Table 1. Using the WT gene of Bst DNA pol LF as template, the Restriction Free (RF) cloning PCR method [17] was applied to construct the mutated genes with primer pairs shown in Table 1. The Bst DNA pol LF proteins were expressed with C-terminal 6x His-tag promoting proteins purification. All purified proteins (WT and mutants) and the commercial Bst 2.0 DNA polymerase were evaluated by SDS-PAGE.

### 2.4. Expression and Purification of the WT and Mutant Derivatives of Bst DNA pol LF

Plasmid pET21a-Bst-LF-H_6_ was transformed into* E. coli* BL21 (DE3) (Novagen, Madison, WI, USA). The recombinant bacteria were induced to express recombinant LF-Bst-H_6_ by adding isopropyl-*β*-D-thiogalactopyranoside (IPTG) at a final concentration of 1 mM to a culture with OD_600_ of approximately 0.6–0.8 and incubating at 37°C for 6 h. Cell culture (1 L) was harvested by centrifugation at 5000 ×g for 30 min. The cell pellets were resuspended in 40 mL 1x PBS buffer (50 mM Tris-HCl and 300 mM NaCl) and lysed with 450 sonication pulses (400 W, 3 s with a 5 s interval) cooled in ice water bath. The suspension was centrifuged (11,000 ×g at 4°C for 30 min) and passed through a 0.22 *μ*m filter followed by applying to a 5 mL HiTrap^FF^ chelating HP column. The target protein Bst-LF-H_6_ was purified using the standard nickel affinity chromatography procedure and washed with 5 column volumes each of 50 mM, 100 mM, 150 mM, and 200 mM imidazole in column buffer (20 mM Tris-HCl, 500 mM NaCl, and 20% glycerol, pH 8.0) followed by gel-filtration on Bio-Gel P-6 (Bio-Rad Laboratories, Inc.) according to the manufacturer's instructions. Bst DNA pol LF samples were collected and stored in buffer (20 mM Na-phosphate buffer, pH 7.4, 50 mM NaCl). All fractions were dissolved in SDS sample buffer and loaded on 12% (w/v) sodium dodecyl sulfate polyacrylamide gel electrophoresis (SDS-PAGE) and stained with Coomassie Blue. The concentrations were determined by BCA Protein Assay Kit (Sangon, Shanghai, China). Fractions of the elute were stored in store buffer (20 mM Tris-HCl, 50 mM NaCl, and 20% glycerin, pH 8.0) at −80°C.

### 2.5. Enzymatic Activity and Protein Assays

The VP1 gene at genome positions (nucleotides 2978 to 3248) in EV71 subgenotype C4 isolate (GenBank accession number GQ279370.1) from Chinese Center for Disease Control was selected as reference virus. The primers for the IMSA assay was designed with the aid of Primer Explorer V4 [8] shown in Table 2 and synthesized by Huada Gene Co., Ltd. (Shenzhen, China). The optimal condition of IMSA assay was carried out in a 25 *μ*L volume containing the components: 12.5 *μ*L of 2x isothermal reaction mixture consisted of reaction buffer and dNTPs (Deaou Biotechnology, Guangzhou, China), 1.0 *μ*L of each primer (DsF and DsR, 5.0 mM; FIT and RIT, 20.0 mM; and SteF and SteR, 40.0 mM), 0.3 *μ*L of EV71 DNA template, and 1.0 *μ*L of commercial Bst 2.0 DNA polymerase (8 U/*μ*L; New England Biolabs, MA, USA) as positive control while the other tubes contained equal amounts of WT or mutant proteins, finally adding ddH_2_O up to 25 *μ*L in reaction tubes. The reactions were evaluated in two different ways. The first assay was the visual IMSA assay by adding 1.0 hydroxynaphthol blue (HNB) dye to the mixture before amplification, and the other assay was performed in a Deaou-308C constant temperature fluorescence detector (Deaou Biotechnology, Guangzhou, China) with the addition of 1.0 *μ*L diluted SYTO®9 fluorescent nucleic acid (Life Technologies, Gaithersburg, USA). The DNA amplification was performed at 63°C for 60 min and then terminated by heat at 80°C for 2 min.

### 2.6. *k*_cat_ Studies of Bst DNA Polymerase

In recent years, various studies have applied a rapid and sensitive assay with HPLC to separate and quantify low concentration of dNTPs [18, 19]. In this study, the ion-pair reversed phase HPLC method was used to determine the kinetic parameter *k*_cat_ of Bst DNA polymerase by calculating the mass decrement of dCTP. The reaction mixture with high purity containing 4 acid-soluble compounds, dCTP, dGTP, dTTP, and dATP, was prepared and fixed at 100 *μ*M. IMSA assay was performed with the methods of enzymatic activity at total volume 75 *μ*L. The reaction volume was diluted into 750 *μ*L with ddH_2_O to prepare the HPLC sample. Chromatography was performed with Symmetry C18 3.5 *μ*m (4.6 × 150 mm) column (Waters, Milford, USA) equipped to a Nova-Pak C18 Sentry Guard Column with UV detection at 254 nm. The mobile phase was delivered at a flow rate of 1.0 mL/min, with the following gradient elution program: solvent A (10 mM tetrabutylammonium hydroxide, 10 mM NaH_2_PO_4_, and 0.25% MeOH, pH 6.9)/solvent B (5.6 mM tetrabutylammonium hydroxide, 50 mM NaH_2_PO_4_, and 30 MeOH, adjusted to pH 7.0) (v/v), 40/60 at 0 min, 60/40 at 30 min, and 60/40 at 60 min. Polymerization efficiency was measured in triplicate at dCTP concentration and *k*_cat_ (total dNTPs sec^−1^) was calculated to give turnover numbers of nucleotides incorporated per sec.

## 3. Results

### 3.1. Gene Cloning of WT and Mutant Derivatives of Bst DNA pol LF

**Figure 1:**
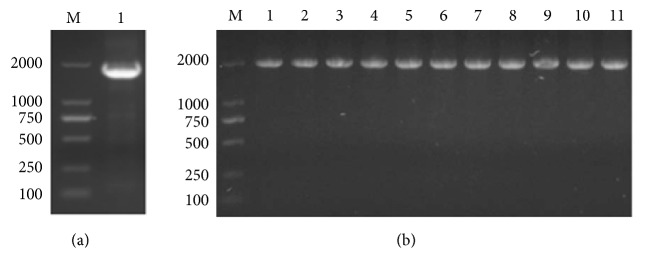
Identification of recombinant plasmids by colony PCR. (a) M: 2 kb ladder marker; 1: WT of Bst DNA pol LF gene; (b) M: 10 kb ladder marker; 1: WT of Bst DNA pol LF gene; 2: LF mutant D540A; 3: LF mutant D540E; 4: LF mutant G310A; 5: LF mutant G310L; 6: LF mutant R412A; 7: LF mutant R412E; 8: LF mutant K416A; 9: LF mutant K416D; 10: LF mutant G310A-D540E; 11: LF mutant G310L-D540E.

To understand the conserved amino acid in dNTPs binding and polymerization region of Bst DNA pol LF, 4 amino acid residues mutations of Gly^310^, Arg^412^, Lys^416^, and Asp^540^ were introduced on the surface of the polymerase cleft by site-directed mutagenesis. Bands corresponding to 1758 bp WT of Bst DNA pol LF and 10 genes of site-directed mutants were detected on 1.5% (w/v) agarose gel by colony PCR of recombinant plasmids (Figure 1), demonstrating that WT of Bst DNA pol LF and mutant genes were successfully inserted into pET21a vectors, respectively.

### 3.2. Expression and Purification of the WT of Bst DNA pol LF

**Figure 2:**
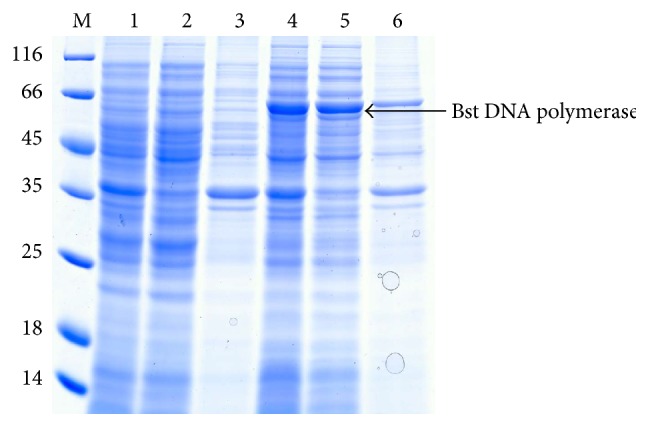
SDS-PAGE analysis of the WT of Bst DNA pol LF. M: protein ladder marker shown in kDa on the left sides of panels; 1: uninduced whole cell sample; 2: supernatant fraction of uninduced sample; 3: pellet fraction of uninduced sample; 4: whole cell sample after induction for 6 h; 5: supernatant fraction after induction for 6 h; 6: pellet fraction after induction for 6 h. Corresponding position of Bst DNA pol LF was marked by black arrow.

After OD_600_ of culture reached mid-logarithm time,* E. coli* BL21 (DE3) cells containing C-terminal 6x His-tagged WT of Bst DNA pol fusion protein were induced by adding 1 mM IPTG and cultured at 37°C for 6 h. Recombinant target protein could be observed by Coomassie Blue staining as a prominent band with an apparent molecular mass of 64 kDa after separation by SDS-PAGE (Figure 2). The recombinant WT of Bst DNA pol LF fusion protein in whole cell lysate was 15.4% and 75.9% of target protein was in soluble fraction. The purity was determined by the gray scale scanning analysis with Bio-Rad Quantity One image quantification system. Purification using affinity chromatography is described in the experimental procedures.

### 3.3. Expression and Purification of the Derivatives of Bst DNA pol LF

**Figure 3:**
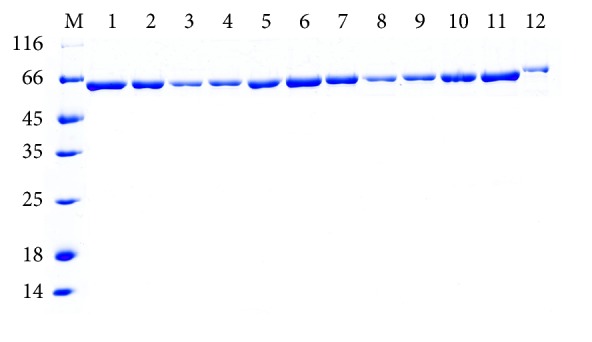
SDS-PAGE analysis of recombinant Bst DNA pol LF and mutant enzymes purified by one-step affinity chromatography. M: protein ladder marker shown in kDa on the left sides of panels; 1: WT Bst DNA pol LF; 2: LF mutant D540A; 3: LF mutant D540E; 4: LF mutant G310A; 5: LF mutant G310L; 6: LF mutant R412A; 7: LF mutant R412E; 8: LF mutant K416A; 9: LF mutant K416D; 10: LF mutant G310A-D540E; 11: LF mutant G310L-D540E; 12: commercial Bst 2.0 DNA polymerase.

We constructed eight mutant genes of Bst DNA pol LF with individual amino acid substitutions at the tip of the “thumb” region which is thought to fold over the DNA template. The clones were transferred into* E. coli* BL21 (DE3) to express mutant enzymes at reasonably high level followed by protein purification according to the same procedure of Bst DNA pol LF (Figure 3). There was a clear band in each of the Bst mutant samples at approximately the calculated molecular weight of the enzyme (64 kDa) and purity of all samples prepared using this method was >95%. The purity was determined as described in Section 3.2. Interestingly, from the SDS-PAGE, the molecular weight of the WT of Bst DNA pol LF and the mutant enzymes was a little bit smaller than that of the commercial Bst 2.0 DNA polymerase indicating that the Bst DNA pol LF in this study was not similar to market available Bst DNA polymerase.

### 3.4. IMSA Assay

Using the method of visual IMSA to analyze the WT and mutant enzyme activity, positive reactions in the tubes displayed a sky blue color, while the negative reactions displayed violet color. The final result of fluorescence values showed that commercial Bst 2.0 DNA polymerase displayed enzyme activity. Mutant D540E has polymerization efficiency values identical to WT DNA polymerase whose curve is two minutes earlier than commercial Bst 2.0 DNA polymerase. Mutant derivatives G310A and G310L showed three minutes earlier peak than WT DNA polymerase which demonstrated Gly^310^ displaced by Ala or Lys having significantly increased polymerization efficiency. In contrast, other mutants such as double residues substitution G310A-D540E and G310L-D540E and the replacement of Asp^540^ by Ala, Arg^412^ by Ala or Glu, and Lys^416^ by Ala or Asp resulted in almost complete inactivation of the polymerase activity. The mutation D540E does not affect the DNA polymerase activity of the enzyme, and presumably there is less distortion of the molecule interaction than the other mutations.

### 3.5. Kinetic Parameter* k*_cat_ Study of Bst DNA pol LF

**Figure 4:**
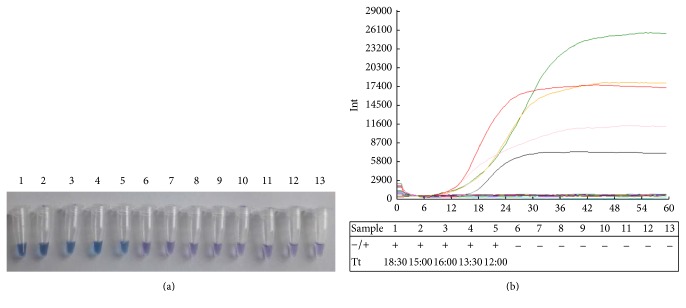
Visual IMSA assay and sensitivity evaluation of IMSA assay to test EV71. (a) Visual detection was performed with IMSA assay by adding HNB dye prior to amplification procedure. The color of sky blue demonstrates positive reactions while the color of violet demonstrates negative reactions. The number of the tube indicates IMSA reaction, respectively, as follows: 1: commercial Bst 2.0 DNA polymerase; 2: WT of Bst DNA pol LF; 3: LF mutant D540E; 4: LF mutant G310A; 5: LF mutant G310L; 6: LF mutant D540A; 7: LF mutant R412A; 8: LF mutant R412E; 9: LF mutant K416A; 10: LF mutant K416D; 11: LF mutant G310A-D540E; 12: LF mutant G310L-D540E; 13: negative control. (b) Fluorescence signals on real-time PCR instrument. Fluorescence values and curves were evaluated with Deaou-308C constant temperature fluorescence detection equipment. The reaction order in (b) table was arranged the same as tubes number in (a). The sign of “+” indicates positive reactions while “−” indicates negative reactions. Reactions 1–5 were able to amplify VP1 gene to detect EV71. The curves in different colors represent distinct proteins in IMSA reaction. Curve in black and “reaction 1” represent commercial Bst 2.0 DNA polymerase. Curve in green and “reaction 2” represent WT of Bst DNA pol LF. Curve in orange and “reaction 3” represent LF mutant D540E. Curve in pink and “reaction 4” represent LF mutant G310A. Curve in red and “reaction 5” represent LF mutant G310L.

**Figure 5:**
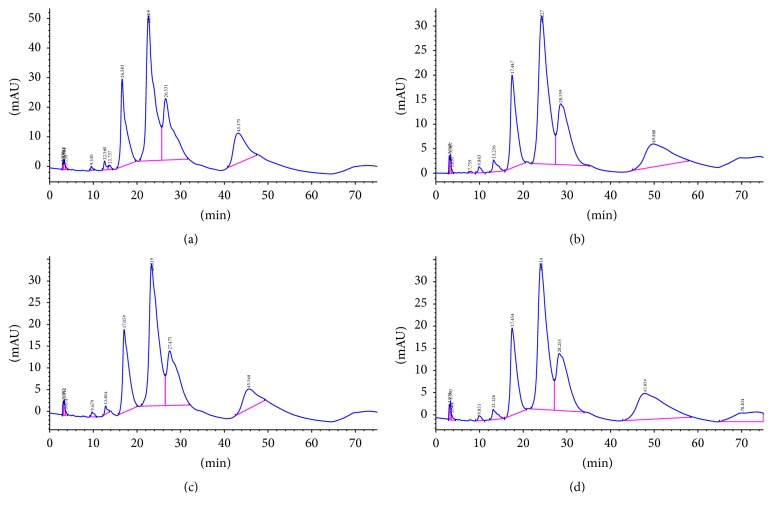
HPLC analysis of polymerization efficiency of Bst DNA polymerases in IMSA assay. (a) Negative control: retention time of dCTP is 16.583 min and the peak area is 2459.42; (b) the LF mutant G310L: retention time of dCTP is 17.447 min and the peak area is 1781.62; (c) Bst DNA pol WT: retention time of dCTP is 17.059 min and the peak area is 1840.69; (d) commercialized Bst 2.0 DNA polymerase: retention time of dCTP is 17.454 min and the peak area is 1941.52.

The steady-state kinetic parameter *k*_cat  (dCTP)_ was measured in the presence of saturating concentration of dNTP as shown in Figure 5. The qualities of primers and templates were sufficient in IMSA assay in which standard consumption of dCTP by HPLC analysis was used to calculate kinetic parameter *k*_cat  (dCTP)_ of enzymes in positive reaction of visual IMSA assay and sensitivity evaluation assay (WT, commercial Bst DNA pol, and mutant derivatives G310L, G310A, and D540E). The linear relationship between the contents of the dCTP and the chromatographic peak area was investigated and described by the linear equation *y* = −174.22 + 26309.69*x* (*R* ^2^ = 0.997). Kinetic parameters *k*_cat  (dCTP)_ of protein samples in 5 positive reactions in Figure 4 were calculated based on the linear equation. All of these WT and mutants exhibit different *k*_cat  (dCTP)_ (S^−1^) from commercial Bst DNA pol and the results were as follows: G310L: 0.286 ± 0.046, WT: 0.239 ± 0.042, commercial Bst DNA pol: 0.201 ± 0.061, G310A: 0.243 ± 0.042, and D540E: 0.186 ± 0.039. In conclusion, the turnover numbers for dCTP in IMSA assay catalyzed by Bst DNA pol and mutant derivatives followed the order: G310L > G310A > WT of Bst DNA pol > D540E > commercial Bst 2.0 DNA pol. Mutations at Gly310 caused an increase in *k*_cat_ implying that this amino acid may play an important role in catalyzing DNA polymerization.

## 4. Discussion

HFMD characterized by fever, sore throat, ulcers in the oral cavity, and rashes on the bottoms of the feet and the palms has been a global common infectious disease caused by a variety of enteric viruses [20]. Currently, large-scale outbreaks of HFMD predominantly characterized by EV71 infection, a nonenveloped and positive-stranded RNA virus, have become a serious public health issue in China, therefore raised the widely public attention, and constituted financial burden of government health care system [21]. However, there is neither an effective vaccine nor any valid antiviral medicine for EV71 infection [22]. Therefore, it is urgent to detect acute infectious HFMD; the earlier the diagnosis, the easier the prevention of virus transmission to other children and infants to avoid public health emergencies [23]. The detection limit of LAMP assay is not particularly appropriate for assessing low viral load specimens in the early phase of HFMD, resulting in a high risk of false-negative diagnosis rate [24]. In comparison with the traditional confirmatory diagnosis, as a new type of isothermal amplification technology, IMSA assay is a novel, real sensitive, and promising method based on visual inspection of fluorescence or color under natural light changes from negative to positive samples [25]. To rapidly test human EV71, visual IMSA assays using the highly conserved regions of VP1 gene were developed to devise the corresponding IMSA primers. The detect results indicated that the normal six-primer combination in the IMSA assay possessed high specificity and sensitivity.

This polymerase shares 97.5% sequence identity with the previously identified DNA polymerase I of* Bacillus stearothermophilus* [14]. Thermostable DNA polymerase I used in commercial detection kits has been well protected by patents leading to relatively high price and restricted application in China. We cloned and recombinantly expressed the polymerase domain from* Geobacillus stearothermophilus* which is topologically similar to the corresponding domains of the Klenow fragment consisting of a right hand *α* helix with three subdomains: “palm,” “fingers,” and “thumb” to bind duplex DNA [26, 27]. Slight changes in the conformations of these domains may affect substrate binding or the polymerization efficiency [28]. The universally conserved Asp^540^ in Bst DNA pol LF of binding with 3′-hydroxyl of the primer strand to form a hydrogen bond is essential for activity of catalytic polymerization of dNTPs [29]. Asp^540^ performs a key role in catalyzing the formation of the phosphodiester bond. The presence of Mg^2+^ ion which coordinated with Asp^540^ kept Bst DNA pol LF staying in the closed conformation. In the absence of metal ion, Asp^540^ forms a hydrogen bond with the 3′-hydroxyl which subsequently interacted with the introduced Mg^2+^ ion and changed the position of sugar pucker at the 3′-terminus of the primer strand to provide sufficient space for attacking the *α*-phosphate of the nucleotide [30]. The preinsertion site of Bst DNA pol is highly conserved and Gly^310^ at the end of the O helix involved in forming part motif B in the closed polymerase-DNA-dNTP ternary complexes which controlled the template position throughout every catalytic cycle [9]. Gly^310^ also participated in binding with the center of a helix-loop-helix motif where the template rotated 90 degrees along with the template backbone [31]. Several mutant derivatives of Bst DNA pol LF based on Asp^540^ and Gly^310^ in Bst DNA polymerase were designed and produced to improve the efficiency of DNA polymerization. The *k*_cat_ values of positive mutants G310L and G310A were 42.2% and 20.8% higher than that of the commercial Bst DNA polymerase, respectively. D540E is the other positive mutant in which the *k*_cat_ value is similar to that of commercial Bst DNA polymerase; therefore, substitutions at the active site Gly^310^ and Asp^540^ were made simultaneously to improve efficiency of DNA polymerization. It was disappointing that most of the substitutions were with significant loss of DNA polymerase activity. The dramatic changes in mutants G310A-D540E and G310L-D540E highlight that Gly^310^ and Asp^540^ have a profound effect on stabilizing the catalytic site of Bst DNA polymerase. In this situation, one mutation may be sufficient to alter the substrate polymerization, but more site-directed mutagenesis is detrimental to the stability or activity of Bst DNA polymerase by destroying hydrogen bonds to water molecules or other highly conserved residues (Arg^325^, Glu^368^, Gln^507^, and His^539^) of polymerase active site that participates in the network of hydrogen bonds and electrostatic interaction [29]. All of these residues have significant effect on substrate binding or catalysis when they were mutated. Adopting optimal reaction conditions, the efficiency and sensitivity of Bst DNA polymerases in IMSA assay were evaluated with the artificial template. The WT or commercial Bst DNA polymerase was chosen as parallel tests. Modified Bst DNA polymerase (G310L) displayed higher efficiency of DNA amplification than the WT and commercial samples.

In summary, the modified Bst DNA polymerase employed in IMSA technology, on the one hand, breaks application restrictions of the patent in commercial kit in China; on the other hand, it improved the understanding of the three-dimensional structure of Bst DNA polymerase and the mechanism of DNA amplification. This study has established a fast, simple, accurate, and sensitive diagnostic method for rapidly detecting HFMD associated with EV71 which provides an efficient way for quick identification, especially for the primary diagnostics setting in rural and underdeveloped areas.

## Figures and Tables

**Table 1:** Oligonucleotides used in substitution mutagenesis for 10 residues of Bst DNA pol LF.

Mutagenesis	Primer name	Sequence
G310A	F (5′-3′)	GTGGTGCACCCCGTGACG**GCG**AAAGTGCACACGATGTTCAATCAG
	R (5′-3′)	CTGATTGAACATCGTGTGCACTTT**CGC**CGTCACGGGGTGCACCAC
G310L	F (5′-3′)	GTGGTGCACCCCGTGACG**CTC**AAAGTGCACACGATGTTCAATCAG
	R (5′-3′)	CTGATTGAACATCGTGTGCACTTT**GAG**CGTCACGGGGTGCACCAC
D540A	F (5′-3′)	CGCCTGTTGCTGCAAGTGCAT**GCA**GAACTGATTTTGGAGGCGCCGAAAGA
	R (5′-3′)	CTCTTTCGGCGCCTCCAAAATCAGTTC**TGC**ATGCACTTGCAGCAACAGGCG
D540E	F (5′-3′)	CGCCTGTTGCTGCAAGTGCAT**GAA**GAACTGATTTTGGAGGCGCCGAAAGA
	R (5′-3′)	CTCTTTCGGCGCCTCCAAAATCAGTTC**TTC**ATGCACTTGCAGCAACAGGCG
R412A	F (5′-3′)	CGAAGAAGACGTGACAGCCAACAT**GCA**CGCCAAGCGAAGGCCGTCAATT
	R (5′-3′)	AATTGACGGCCTTCGCTTGGCG**TGC**CATGTTGGCTGTCACGTCTTCTTCG
R412E	F (5′-3′)	CGAAGAAGACGTGACAGCCAACATG**GAA**CGCCAAGCGAAGGCCGTCAATT
	R (5′-3′)	AATTGACGGCCTTCGCTTGGCG**TTC**CATGTTGGCTGTCACGTCTTCTTCG
K416A	F (5′-3′)	GTGACAGCCAACATGCGCCGCCAAGCG**GCA**GCCGTCAATTTTGGCA
	R (5′-3′)	TGCCAAAATTGACGGC**TGC**CGCTTGGCGGCGCATGTTGGCTGTCAC
K416D	F (5′-3′)	GTGACAGCCAACATGCGCCGCCAAGCG**GAT**GCCGTCAATTTTGGCA
	R (5′-3′)	TGCCAAAATTGACGGC**ATC**CGCTTGGCGGCGCATGTTGGCTGTCAC
G310A-D540E	F (5′-3′)	GTGGTGCACCCCGTGACG**GCG**AAAGTGCACACGATGTTCAATCAG
	R (5′-3′)	CTGATTGAACATCGTGTGCACTTT**CGC**CGTCACGGGGTGCACCAC
G310L-D540E	F (5′-3′)	GTGGTGCACCCCGTGACG**CTC**AAAGTGCACACGATGTTCAATCAG
	R (5′-3′)	CTGATTGAACATCGTGTGCACTTT**GAG**CGTCACGGGGTGCACCAC

Underlined sequences indicate the mutant codons.

**Table 2:** Oligonucleotides used in IMSA assay.

Primer name	Sequence
DsF-EV71	5-ACCATTGATAAGCACTCGCAGGGTCAAGCTGTCAGACCCTCC-3
DsR-EV71	5-GAACACAAACAGGAGAAAGATCTTGTGAGAACGTGCCCATCA-3
FIT-EV71	5-TCCGAATGTGGGATATCCGTCATAAGTTTCAGTGCCATTCATGTC-3
RIT-EV71	5-TTATGACGGATATCCCACATTCGGAAGGACATGCCCCGTATT-3
SteF-EV71	5-GAACACAAACAGGAGAAAGATCTTG-3
SteR-EV71	5-ACCATTGATAAGCACTCGCAGG-3
